# *Wh-*Movement, Islands, and Resumption in L1 and L2 Spanish: Is (Un)Grammaticality the Relevant Property?

**DOI:** 10.3389/fpsyg.2020.00395

**Published:** 2020-03-24

**Authors:** Sílvia Perpiñán

**Affiliations:** Department of Applied Linguistics, Universitat Internacional de Catalunya, Barcelona, Spain

**Keywords:** wh-movement, islands, spanish, processibility, L2 learners, resumptive pronoun

## Abstract

This study reflects on the meaning of the results of a self-paced grammaticality judgment task that tested island configurations (with gaps and resumptive pronouns) in L1 and L2 speakers of Spanish. Results indicated that resumptive pronouns do not rescue extractions from islands, as traditionally assumed in grammatical theory, and propose that islands are essentially an interpretative or processing matter, and not only a grammatical one, as in Kluender (1998). This study further challenges the L2 studies that proposed that L2 learners are fundamentally different from native speakers because they usually fail to reject island configurations, and shows that L2 learners are sensitive to the same processing and interpretative mechanisms that native speakers employ to parse island configurations. Generally speaking, this study proposes that apparent purely syntactic restrictions such as extractions from islands might not depend on their grammatical formation, but on other relevant factors such as plausibility, embedding, and processability, which together with grammatical well-formedness configure a more holistic and useful notion of linguistic acceptability.

## Introduction

The concept of grammaticality has been of vital importance in the development of the field of modern linguistics, particularly since Chomsky’s influential books, *Syntactic Structures* (Chomsky, 1957) and *Aspects of the Theory of Syntax* (Chomsky, 1965). The study of what is possible and, crucially, what is not possible in a language has allowed us to deepen our knowledge on particular and universal properties of linguistic systems. In the field of Second Language Acquisition from a Generative Perspective (GenSLA), the notion of grammaticality has also been essential in order to determine the nature of interlanguage grammars and to describe the implicit linguistic knowledge of a second language learner. Generative linguistics generally assumes that Universal Grammar (UG), which is domain-specific, takes care of the breach left between what is acquired through input and what is deduced by general cognition. Much of the debate in GenSLA during the 80s and 90s revolved around whether interlanguage grammars and native grammars are fundamentally similar or fundamentally different, and whether the former could access UG after the critical period of acquisition (for a summary, see White, 1989, 2003). Constraints on *wh*-movement, i.e.: Subjancency, have been taken as the ideal case to test the accessibility of interlanguage grammars to UG since they typically illustrate the poverty-of-the-stimulus problem. Islands are not present in the L2 input or taught in a classroom setting, and one can find L1 languages in which *wh-*movement does not operate. Therefore, these L2 learners cannot rely on L1 knowledge or direct L2 input to know the restrictions on *wh*-movement. The logic goes as follows: if we can show that these L2 learners whose L1 does not have *wh-*movement obey the subjacency constraints that regulate *wh*-movement, then we can conclude that their knowledge must come from UG (but see Pearl and Sprouse, 2013 for a different explanation). With this in mind, researchers have traditionally employed Grammaticality Judgment Tasks (GJT) as a technique to tap into the underlying grammatical representation of (non-)native speakers, which crucially affords us to test both possible and impossible sentences. This study reflects on the concept of grammaticality in both native and non-native grammars, and on how it has been used to argue for or against the accessibility to UG by adult second language learners, a central issue in GenSLA. It further questions the assumed (un)grammaticality of certain complex structures, such as island configurations or islands rescued by resumptive pronouns (RPs), and particularly, its syntactic nature. Heestand et al. (2011) already proposed that resumption does not necessarily rescue islands in English, but the application of these recent ideas in the second acquisition research has been very scarce, and L2 data that support these claims are practically inexistent. Likewise, a similar study on L2 Spanish is missing. Moreover, the acquisition of oblique relative clauses is widely unexplored, particularly in real-time use, in which processing resources might be compromised and resumption as a last resort could be favored (McCloskey, 1990). The present study aims to fill these gaps in the literature.

## The Linguistic Phenomenon: Islands and *Wh*-Movement

*Wh*-movement is an extensively studied topic in generative linguistics, especially since Chomsky (1977) proposed that the transformation involved in questions, relative clauses, comparatives, or easy-to-please constructions could be reduced to the general “wh-movement” transformation, a successive cyclic movement to COMP. Later, Chomsky (1981)’s Government and Binding framework presented wh-movement as an instance of a more general transformation: *move* α, regulated among others, by the Subjacency Principle (Chomsky, 1986), which basically controls how far a *wh-*phrase can move, and is supposed to be universal. The original subjacency condition posited that “a constituent may not move over more than one bounding category at a time” (Chomsky, 1973). Even though the concept of bounding nodes may have changed as linguistic theory has evolved, the idea is that Subjacency explains the contrast between (1b) and (2b) because in (1b), the *wh*-word crosses one bounding node at a time, first the IP and then the CP, with successive cyclic movement; whereas in (2b), the first movement crosses one bounding node, -the IP-, but it crosses two in the second movement, the CP and the DP, which renders the sentence ungrammatical. This observation led to propose that complex DPs, in this case a Relative Clause, are “islands” [in Ross’ (1967) terminology] from which a *wh-*word cannot be extracted. Examples from Belikova and White (2009):

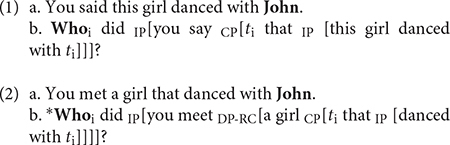

In the last 20 years, there has been a significant amount of experimental work that aims to explain the source of the unacceptability of *island effects* (see Sprouse and Hornstein, 2013 for a summary), a classic issue in syntactic theory since Ross (1967). Much has been debated regarding whether islands are a grammatical entity or a parsing one; that is, whether the structure-building constraints that restrict *wh-*movement from certain domains are a syntactic grammatical representation in the cognitive system (a position usually termed as “grammatical theories”, Phillips, 2013) or whether islands effects arise as a result of a processing failure or processing limitation, an epiphenomena that comprehends multiple factors such as semantic anomaly, processing difficulty, etc. (“resource-limitation theories,” Kluender, 1991, 1998; Kluender and Kutas, 1993; Hofmeister et al., 2013; Kluender and Gieselman, 2013). This dichotomy closely ties grammatical theories with real-time language processing (Phillips, 2006; Lewis and Phillips, 2015) and echoes a fundamental controversy in SLA theories when trying to explain the cause of non-convergence in L2 learners (representational vs. computational accounts, Hopp, 2007, 2009; Slabakova, 2009; Perpiñán, 2015). That is, whether L2 learners have permanent representational deficits, probably due to a partial (Hawkins and Chan, 1997), or no access to UG (Bley-Vroman, 1990, 2009; Meisel, 1997), or whether L2 learners are not able to process the language as efficiently or with the same syntactic detail as native speakers (Clahsen and Felser, 2006). Sprouse et al. (2012) even consider, although do not defend, a third option to explain island effects in L1, which is a combination of the grammatical and reductionist accounts, termed *grounded theories*. Grounded theories assume that island effects are caused by grammatical constraints that have been grammaticized over time because if these structures were generated, these would be difficult to parse. To summarize, the island debate in native languages is an especially multifactorial puzzle that adds to the unresolved challenges in the study of L2 knowledge and its processing, the current debates in the field of SLA.

Ever since Subjacency was put forward as a grammatical explanation of island violations, it has been studied widely in the SLA field as it allows us to make pertinent predictions regarding the role of UG in the interlanguage grammar. If L2 acquisition is constrained in all its instances by UG, then, all possible L2 interlanguage grammars should obey universal principles, including the Subjacency Principle, regardless of the learners’ L1, the target language, and their *wh*-movement properties. Island configurations have been typically used as a test for syntactic movement: if an extraction requires syntactic movement, then, that construction will be ungrammatical if it is extracted from an island. If, on the contrary, a constituent is apparently extracted from an island and the derivation is not ruled out, then it is assumed that there was no movement involved. In that case, we would say that there was not an extraction *per se*, but that the constituent was base-generated and bound somehow with its antecedent.

Traditionally, an assumed way to rescue an island violation is by introducing a resumptive pronoun (Ross, 1967; Kroch, 1981; McCloskey, 1990; Shlonsky, 1992). According to McCloskey (2007), we can group three types of languages that employ resumptive pronouns (RPs) differently; in this study we are concerned with two of these types, Type I and Type III. Type I languages would allow free variation of RPs and gaps; inside an island though, only resumptive pronouns can appear. This is the case of Lebanese Arabic as described by Aoun et al. (2001), and we will assume that it is also the case of Moroccan Arabic, the variety that concerns us in this experiment. However, Shlonsky (1992) argues that the use of (true) resumptive pronouns in Hebrew and Palestinian respond to a last resort strategy, meaning that they are used when operations general to Universal Grammar are blocked. According to this author, the use of resumptive pronouns is a language-specific rule that must apply whenever movement is not available, and it is not optional. This might be true for direct object relative clauses, but Arabic prepositional relative clauses present both strategies, movement and resumptive pronouns, as explained below. Type III languages are those that present “intrusive pronouns” (Sells, 1984), which are not a true pronoun or syntactically active resumptive (Asudeh, 2012) as it does not alternate with gaps and is not island-sensitive. We are assuming that this is the case for both English and Spanish.

Recently, there have been different proposals to explain RPs, and their power (or lack thereof) to ameliorate illicit island extractions has been seriously questioned. In a nutshell, syntactic and off-line data seem to indicate that RPs do improve island violations, whereas psycholinguistic data have failed to find strong evidence that supports this claim. For instance, Alexopoulou and Keller (2007), as well as Heestand et al. (2011), and Polinsky et al. (2013), in a series of experimental studies testing different types of island configurations with and without pronouns, found that when extracting from an island, strong or weak, the resumptive structure was never judged “more grammatical” than its gapped version. Polinsky et al. (2013) proposed, then, that RPs do not establish an A’ binding relationship, but a co-referential one. That is, RPs in English do not obey syntactic considerations but discourse-pragmatic ones, as they are considered anaphors. This was found in both on-line and off-line acceptability judgments. Likewise, McDaniel and Cowart (1999) found in an acceptability judgment task that native speakers of English did not prefer the resumptive pronoun over the trace structure in contexts in which the movement operation was illicit, i.e.: in island configurations, but they did prefer them in violations of conditions on representation. This made McDaniel and Cowart (1999) conclude that resumptive pronouns do not repair violations of the derivation (movement violations), and that they are spell-outs of traces. On the other hand, Ackerman et al. (2018), using several off-line forced-choice binary tasks, found that speakers of English strongly preferred RPs in island contexts, concluding that RPs indeed ameliorated island-violating sentences and questioned the assumed ungrammaticality of object-extracted resumptive pronouns in English.

More recently, in a further attempt to explain the syntactic and psycholinguistic nature of resumptive pronouns, Morgan and Wagers (2018) found a negative correlation between the acceptability of a gap structure and the production of RPs: as the acceptability of a structure with a gap decreases, the frequency of production of RPs in that structure increases. This result closely relates the production and comprehension domains, and indirectly rejects the idea that the production and the comprehension systems may consult different grammars, as Ferreira and Swets (2005) have suggested. Likewise, Chacón (2019) proposes that when speakers (comprehenders) try to resolve a filler-gap dependency, they do it preferably through a gap, which needs to be maintained in working memory over time. If working memory is strained though, then resumption becomes more acceptable. Thus, inasmuch as island configurations might suppose a burden for working memory, then they are a good host for resumption. To sum up, as this condensed review of studies dealing with resumptive pronouns in island configurations has shown, the paradox over RPs, —why are they produced by native speakers who rate them as unacceptable? —, as well as their nature —are they a processing entity or a syntactic one? —, are still open questions in the field, and even more so in SLA.

The general purpose of this study is to describe the nature of the Spanish interlanguage grammar of English and Arabic speaking learners by exploring *wh-*movement knowledge and its constraints. Ultimately, we want to determine whether L2 learners’ knowledge is different or similar to that of a native speaker. With this in mind, we collected written production data of prepositional relative clauses as well as online grammaticality judgments on extractions from island configurations, in both conditions, with a gap or a resumptive pronoun. In turn, the acceptability data from our control group, the native speakers’ data, will also serve us to reflect on the supposed (un)grammaticality of certain constructions, on the components that configure a grammatical judgment, and more in particular on the theory of *wh-*movement in L1 and L2. The following paragraphs will be devoted to explaining the three different strategies that prepositional relative clauses present in (Moroccan) Arabic, English and Spanish. The three possible syntactic strategies are Pied-Piping, Preposition Stranding, and Resumption.

Arabic, English and Spanish oblique relative clauses can be formed through Pied-Piping, a strategy which consists of moving the obligatory preposition along with the relative pronoun, as in (3). This strategy clearly involves *wh-*movement:

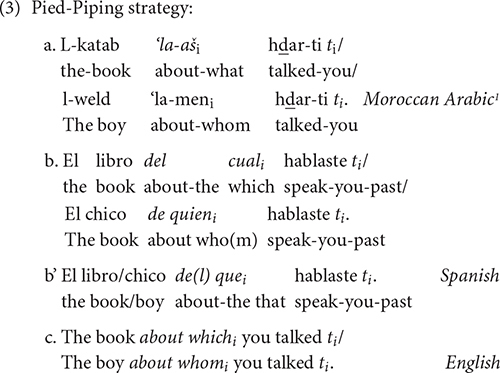
^1^

Moreover, English can leave the preposition dangling in its original position once the displaced constituent has moved; this option is ungrammatical in Spanish and Arabic, as the examples in (4) show, and involves movement:

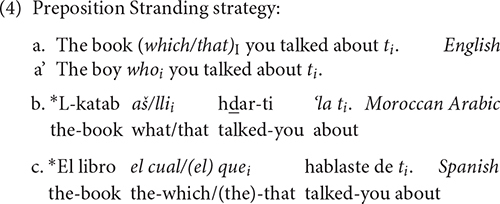

Finally, only Arabic accepts relative clauses with resumptive pronouns in its standard varieties. In fact, it is the most common strategy in standard Arabic, whereas it is ungrammatical or non-standard in English and Spanish, as the contrasts in (5) illustrate. This option in Arabic is not a last-resort strategy, as it could be the case in English or Spanish. In any case, resumptive pronouns appear always with complementizers and not with relative pronouns, as the contrasts among languages in (5) show.

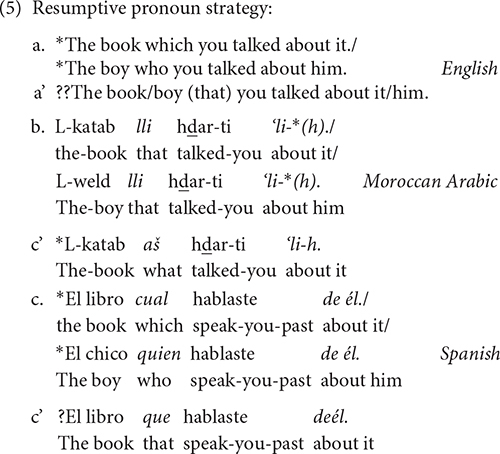

The question that arises here is whether these Arabic resumptive constructions involve movement or binding. The standard analysis for Arabic is that resumption involves binding, and relativization of an argument out of an island configuration does not produce ungrammaticality, as illustrated with Lebanese Arabic in (6a). However, relativization of an adjunct is ungrammatical (6b) and this indicates that there was a violation of subjacency. These data seem to indicate that movement is available at least in some Arabic relative clauses. Aoun and Benmamoun (1998) presented further evidence from reconstruction effects that also points to a movement analysis for some Arabic relatives. Examples from Aoun and Benmamoun (1998):

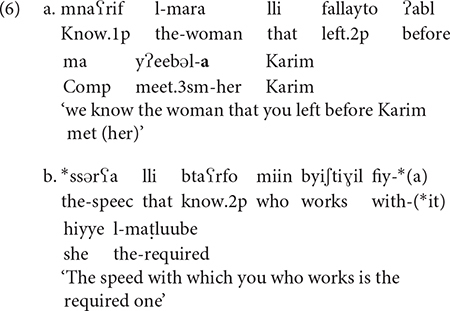

As for Spanish, Suñer (1998) proposed that it is a language that has two types of resumptive pronouns, those optionally inserted in all types of relative clauses (direct and indirect object, prepositional, subject, genitives, locatives), at the level of PF, and those obligatory, used as a last resort, to prevent the structure from an island violation. This type of last resort resumptive pronoun exists in Spanish (7a) and in English (7b), and is the focus of our investigation.

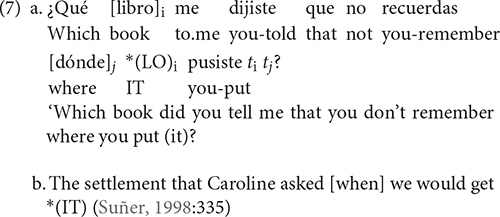

The specific purpose of this study is to first investigate the availability of *wh-*movement in prepositional relative clauses in L2 Spanish, and second, to investigate the grammatical nature of gapped and resumptive islands in L2 learners whose native languages present both *wh-*movement (English and Arabic) and resumptive pronouns (Arabic). Ultimately, we aim to reflect on the concept of grammaticality through acceptability ratings in both native and interlanguage grammars, the reliability of experimental and introspective data, and how these have been used to argue for or against L2 learners’ accessibility to UG.

## *Wh-*Movement and Subjancency in L2 Learners

The availability of *wh-*movement has been the central issue of many studies that discussed accessibility to UG and the differences and similarities between L1 and L2 acquisition (Johnson and Newport, 1991; Hawkins and Chan, 1997; White and Juffs, 1998; among others). In the late 80s, subjancency violations were one of the main arguments for the Fundamental Difference Hypothesis in Second Language Acquisition (Bley-Vroman, 1990; Johnson and Newport, 1991). The early L2 studies on subjacency violations mostly included learners whose L1 does not present overt *wh-*movement, such as Korean or Chinese (Bley-Vroman et al., 1988; Schachter, 1990; Johnson and Newport, 1991; White and Juffs, 1998). For instance, Chinese is a language that does not present overt *wh-*movement, at least with argumental *wh*-movement (Huang, 1982). Johnson and Newport (1991), and Hawkins and Chan (1997) found that the Chinese-speaking learners had problems recognizing subjacency violations in English, a result that made these researchers argue that L2 learners do not have full access to UG, otherwise they would respect the universal principle of subjacency. On the other hand, White and Juffs (1998) found that Chinese speakers with more advanced knowledge of English were accurate at judging these violations, arguing that these L2 learners could indeed access UG. Another general finding in these studies that was later noticed is that performance significantly varied depending on the type of island configuration, L2 learners rejecting strong islands (relative clauses and subjects) more accurately than weak islands (*wh-*islands and noun complements) (Martohardjono, 1993). That is, L2 learners perceived the gradience in grammaticality, which Schwartz and Sprouse (2000) interpreted as an indication of UG access since none of these types of island configurations, weak and strong, are present in the input. This grammaticality asymmetry was accounted for in the revised CED (Huang, 1982; Nunes and Uriagereka, 2000), in which it is stated that subjects and adjuncts are universally islands, as opposed to *wh-*islands, which might be parameterized. Therefore, if L2 learners are not consistent at rejecting weak islands such as *wh-*islands, then these data cannot really inform us about the L2 learners’ accessibility to UG. This is one of the main points raised by Belikova and White (2009), which concluded that, even though islands still constitute a typical poverty of the stimulus scenario, these are now understood to be regulated by computational principles in all languages, and thus, do not speak toward the accessibility to UG, or the difference between L1 and L2 acquisition. The present study reinforces these general conclusions and further questions the assumed grammaticality of certain island configurations.

More recently, L2 studies have implemented on-line methodologies to assess the real-time processing of *wh-*dependencies and island constraints, and to investigate whether L2 learners are able to use syntactic information in real-time processing (Aldwayan et al., 2010; Omaki and Schulz, 2011; Kim et al., 2015; Johnson et al., 2016 a. o.). For instance, Aldwayan et al. (2010) investigated whether Najdi Arabic (a *wh-in situ* language with obligatory resumption) learners of English have the knowledge of syntactic constraints in the processing of *wh-*movement and whether they process these structures incrementally. With a self-paced reading task, they showed that advanced L2 learners are guided by syntactic constraints and posit gaps during incremental language processing, as native speakers do, disproving the Shallow Structure Hypothesis (Clahsen and Felser, 2006). Similarly, Aldosari (2015) found that Najdi Arabic speakers who are learners of English were sensitive to syntactic island constraints on *wh-*movement, and that individual differences such as working-memory capacity did not have an effect on sensitivity to island effects, concluding that islands are not due to limited processing resources but most likely to syntactic constraints. With respect to Spanish-speaking learners of English, Kim et al. (2015) found that Spanish speakers did not keep active a filler-gap dependency in a relative clause island configuration, obeying the same restrictions as native speakers. These authors did not exactly find the same results in Korean learners of English (Korean being a *wh-in situ* language), who seemed to have posited a gap when processing an island configuration even though they showed knowledge of *wh-*movement restrictions in islands in the off-line task. Kim et al. (2015) interpreted these results by proposing that the L1 of the learners influences the L2 learners’ processing. None of these studies, though, directly tackled the issue of resumptive pronouns in SLA, the focus of our investigation.

In order to assess whether L2 learners know the limits of *wh-*movement and the locality constraints that regulate it, first it must be determined that the learners indeed have *wh-*movement in their interlanguage grammars. Some of these studies included *wh-*question formation to show that movement was already mastered, but there is some controversy with this procedure since *wh-*questions can imply topicalization or scrambling, in which movement is not involved. For these reasons, we decided to include relative clause formation in our study. As shown in (3) above, all languages at play in this study can form oblique relative clauses through movement (Pied-Piping); English can also employ Preposition Stranding, another movement structure, and Arabic usually resorts to resumptive pronouns in its relative clauses, a no-movement option. In this study we want to investigate the limits of *wh-*movement in L2 learners whose native language already presents *wh-*movement, an understudied combination. It has typically been the case in the literature that problems rejecting island violations were explained by the lack of *wh-*movement in the L1s of the L2 learners. However, it has not been investigated whether those grammaticality judgments assigned to island configurations were a true reflection of the inability to constrain *wh-*movement, or whether these were measuring a different type of linguistic phenomenon in the L2 learners’ interlanguage. It could be the case that comprehension of island configurations goes beyond the realm of *wh-*movement. This is what we aim to unravel in this study.

Related to the (in)ability to displace *wh-*elements and to create filler-gap dependencies, we also included islands rescued by resumptive pronouns. Resumptive islands in SLA have been hardly investigated, not even in L2 learners whose native language accepts resumptive pronouns in relative clauses, such as the case of Arabic. We believe that, if we want to investigate the nature of island configurations and more particularly the nature of the grammaticality judgments of island configurations in both L1 and L2, resumptive islands need to be included in the experimental design, particularly if one of the languages at play presents resumptive pronouns in its standard variety. Thus, this study is twofold: by focusing on the properties of *wh-*movement in interlanguage grammars and questioning some of the commonly accepted assumptions for island configurations, it aims to generally reflect on the concept of grammaticality in SLA and the theoretical hypotheses that hinge on it. This study has three general research questions (RQ1a, b, c) and two specific research questions (RQ2a, b):

RQ1. a. What do grammaticality judgments tell us about the nature of interlanguage grammars and the native knowledge of a language?
  b. What do judgments on island configurations tell us about *wh-*movement theory?
  c. What do judgments on island configurations tell us about the (in)ability to *wh-*movement in a second language grammar?
RQ2. a. Would L2 learners whose native language already presents some type of *wh-*movement strategy also employ *wh-*movement when forming oblique relative clauses in an L2?
  b. Would L2 learners be able to constrain *wh-*movement appropriately in their second language by rejecting island violations and accepting resumptive islands?

Considering the linguistic phenomenon under investigation and its properties in English and Arabic described in (3–5), we formulate the following hypothesis for the specific research questions:

• H1: Assuming the Full Transfer/Full Access Hypothesis (FT/FAH, Schwartz and Sprouse, 1994, 1996), which postulates full transfer of the L1 and full access to UG in L2 acquisition, if the L1 is fully transferred into the L2 grammar, then, the L2 learners would be able to employ *wh-*movement when forming oblique relative clauses in L2 Spanish. That is, we should not expect L2 learners to have major problems constructing oblique relative clauses through Pied-Piping because this strategy is already present in their L1s.
• H2: Also, assuming the FT/FAH, we could expect some degree of negative transfer, such as Preposition Stranding in English L2 learners’ grammars, and Resumption in Arabic L2 learners’ grammars, especially at early stages of development.
• H3: Finally, if participants already have *wh-*movement in their L1s, then we will find that relative clauses formed as an extraction from an island will be judged as ungrammatical due to subjacency violations. If, on the other hand, they interpret relative clauses through binding and not movement, then these participants will accept ungrammatical extractions out of an island. In both cases, we expect participants to accept extractions from islands rescued by a resumptive pronoun.

## The Study

In order to investigate these questions on the nature of interlanguage and native grammars and *wh-*movement knowledge, we designed a series of tasks. Here, we are reporting the results of two of these tasks: a written production task that elicited relative clauses, and a self-paced grammaticality judgment task with different types of island configurations. The data we are analyzing in this study is part of a series of experiments on the L2 processing and L2 acquisition of relative clauses (Perpiñán, 2010).

### Participants

An initial pool of 20 native Spanish speakers and 116 Spanish learners (L1 English or L1 Arabic) participated in this study. The English-speaking learners (*n* = 81) were college students enrolled at the University of Illinois or at the Knox College at the time of testing (mean age = 21.9). They were all born and raised in the United States, and they were recruited either at intermediate or advanced Spanish courses. Students who used a different language at home (Korean, Polish, Spanish, etc.) and who knew other second languages (as reported on the background questionnaire) were excluded from the data analysis. The Arabic speakers (*n* = 35) were all native speakers of the colloquial Moroccan Arabic variety or “dariℨa”. Native speakers of other languages such as Berber were excluded from the experiment. The Arabic speakers were students of intermediate or advanced Spanish courses either at the Instituto Cervantes or at the language academy “Dar Loughat” in Tetouan, Morocco. Most of them were college students although there were also some civil servants or professionals in the pool (mean age = 25.6). Since it is impossible to find educated participants in Morocco, who have not studied French or have taken courses in French, these subjects are, potentially, L3 speakers of Spanish. However, most of them reported that their knowledge of French was limited and that they felt more comfortable speaking in Spanish than they did in French. The control group consisted of native speakers of Spanish (*n* = 20), 8 males and 12 females, from different dialectal varieties: one Argentinean, one Colombian, one Costa Rican, one Mexican, one Venezuelan, and fifteen speakers of Castillian Spanish. Their mean age at the time of testing was 32.25. All but two were college graduates.

All participants took a proficiency test, which consisted of a slightly modified version of the standardized grammar section of the superior level of the Diploma de Español como Lengua Extranjera (DELE), created by the Instituto Cervantes. In this proficiency test we included six screening items that tested subcategorization knowledge of the prepositional experimental verbs: *hablar de* (to talk about), *depender de* (to depend on), *pensar en* (to think about), *confiar en* (to rely on), *soñar con* (to dream about), *contar con* (to count on). These verbs required a preposition in the three languages we are considering: Spanish, English and Moroccan Arabic. Participants who did not know that these verbs subcategorized a prepositional argument were not invited to continue with the study. After this scrutiny, only 42 L2 learners (21 English speakers/21 Arabic speakers) completed the entirety of the experiment. The participants’ proficiency scores (maximum score 40) were submitted to a one-way ANOVA, and as expected, the results of the ANOVA indicated a significant effect by group *F*(2,59) = 28.74, *p* < 0.001. A *post hoc* Tukey HSD test revealed that the only different group was the control group (*p* < *0.001*), whose mean score was 39.6 (SD.681), with a 99% rate of accuracy. The Arabic (mean score = 25.67, SD = 8.79, 64% accuracy) and English learners of Spanish (mean score = 26.05, SD = 7.32, 65% accuracy) did not differ significantly (*p* = 0.98).

## Task 1: Written Production Task

The purpose of this task was to reveal how productive our participants’ *wh-*movement structures are. Participants were presented with two independent sentences that shared one constituent and were instructed to combine the two sentences, retaining the same meaning while not using the repeated constituent again. The beginning of each new sentence was provided to ensure that the participants used that constituent as the extracted part of the complex sentence. Two examples were provided: the first one demonstrated a prepositional construction and thus, a Pied-Piped relative clause; the second exemplified a transitive construction. The experiment included the 6 target items that required prepositional RCs and 5 items targeting direct object RCs. In this study, we are only interested in the prepositional contexts. Examples are shown in (8) below.

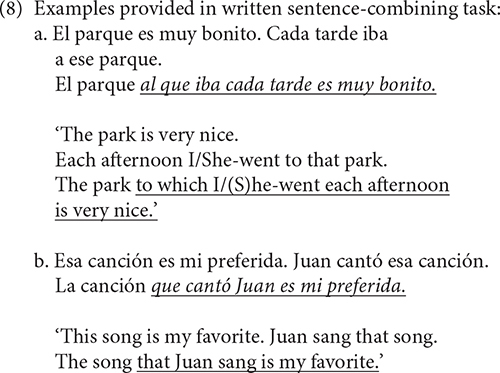

## Results Task 1: Written Production of Relative Clauses

A total of 682 sentences were generated in the written experiment; 372 in the prepositional context are the only ones that we will consider here (see Perpiñán, 2013 for more data). Sentences were coded according to their structure, and frequencies and raw numbers (in parentheses) are calculated for each structure produced; data are displayed in Table 1. In order to compute non-parametric statistics on these categorical data, sentences were coded as “target-like” vs. “non-target-like.” Hence, the baseline for comparison is not the native speakers’ production but the expected construction for each group.

**TABLE 1:** Frequency of constructions produced in written prepositional RC, percentages and raw numbers.

Group	Pied-piping	Null prep	Preposition stranding	Resumptive	No RC	Other	Total
Natives	99.2 (119)	0	0	0.8 (1)	0	0	100 (120)
L2 English	62.7 (79)	15.9 (20)	16.7 (21)	0	3.2 (4)	1.6 (2)	100 (126)
L2 Arabic	46.8 (59)	22.2 (28)	0	20.6 (26)	5.6 (7)	4.8 (6)	100 (126)

Out of the 372 sentences produced, only 257 were target-like. Native speakers behaved as expected, and 99.2% of their sentences were formed through Pied-Piping, but only 62.7% of the English learners’ production and 46.8% of the sentences produced by the Arabic learners were target-like, that is, formed through Pied-Piping.

The percentages alone already seem to indicate that there is a significant difference among the three groups, as the Chi square based on the accuracy of the sentences × groups demonstrates *χ*^2^ (2) = 82.48, *p* < 0.001. Furthermore, the two experimental groups (English vs. Arabic speakers) also differed significantly *χ*^2^ (1) = 6.407, *p* = 0.011 between themselves, as English speakers were more target-like than the Arabic speakers. And since the native group only missed one sentence out of 120, the odd ratios are enormous: English speakers were 70.8 times more likely to be non-target-like than the native group, and in the case of the Arabic speakers, the inaccuracy ratio compared to the control group is up to 135.2. Thus, although Spanish prepositional relative clauses present some difficulties for L2 learners, the target Pied-Piping is nonetheless the most produced construction in both groups.

The deviance from the target structure by the English-speaking learners not only consisted of producing the ungrammatical L1 transferred structure Preposition Stranding, as in (9a), but also a relative clause without the obligatory preposition, a phenomenon termed Null Prep by Klein (1993), such as (9b). The same holds for the Arabic speakers who produced 22.2% of these sentences without the obligatory preposition, as in (10a), and 20.6% of the sentences with the preposition and a strong resumptive pronoun, as in (10b). All instances of RPs appeared with the complementizer “que.”

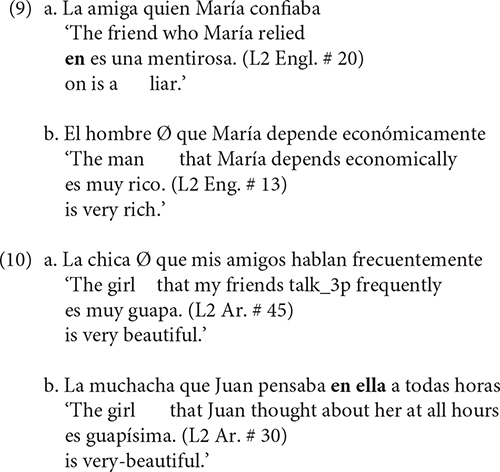

## Task 2: Self-Paced Grammaticality Judgment Task

### Procedure

The self-paced reading task consisted of a total of 84 items followed by a yes/no grammaticality judgment question. Half of the sentences were grammatical, and half ungrammatical. 24 of these sentences were relative clauses (see Perpiñán, 2015), 18 items tested subjacency constraints, our experimental conditions, and the remaining 42 sentences were distracters. Sentences were pseudorandomized so that no token from the same condition would appear consecutively. Participants (the same ones as in the previous task) had to read the sentences in a self-paced, non-cumulative word-by-word display on a computer monitor, using the experimental software Linger. The segments initially appeared as a row of dashes, and participants pressed the space bar on the keyboard to reveal each subsequent word of the sentence. At the end of each sentence, participants had to answer the question “Esta frase, ¿está bien?,” (‘*This sentence, is it ok?’)* and then answer as quickly as possible pressing the keys “F” for *yes* and “J” for *no*. These keys were shown in a different color on the keyboard. Participants received immediate feedback if they responded differently than expected: “!‘Oh, lo siento!” (Oops, I’m sorry). This feedback was mainly included to encourage participants to stay focused on what they were reading. Nevertheless, all participants were instructed to follow their intuition when judging the sentences, regardless of the feedback prompted. In fact, they were warned that the computer was not always right and that it was legitimate not to agree with the computer’s feedback.

### Stimuli

The results of the written production task served us to select the three types of extraction from an island that we included in the GJT: Pied-Piping extraction, Null-Prep extraction, and extraction with a resumptive pronoun in the island configuration. We chose strong islands (if-clauses) since previous literature has shown that weak islands might be parameterized and do not hold in all languages, and that L2 learners are mostly sensitive only to this type of islands. Participants needed to make a judgment about the grammaticality of the sentence as fast as possible. The head of the relative clause was extracted from a strong island, specifically a conditional clause. The relative clause was formed either through Pied-Piping, Null-Prep or Resumption. There were six items per condition, one item per each experimental prepositional verb (*depender, hablar, pensar, contar, soñar, confiar*) (3 × 6 = 18 island-type sentences). To avoid confusion, the pseudorandomization ensured that no island sentence of any type would appear right after another island sentence. Also, and since these were long distance extractions, we made sure that the extracted constituent could not be interpreted as an argument of the antecedent of the conditional clause. For this reason, only intransitive verbs were included in this position such as *dormir* (“to sleep”), *callar* (“to shut up”), or *respirar* (“to breath”).

The control structure was the Pied-Piping island configuration (11). There is no disagreement with respect to the ungrammaticality of this construction since Pied-Piped relative clauses undoubtedly involve wh-movement.

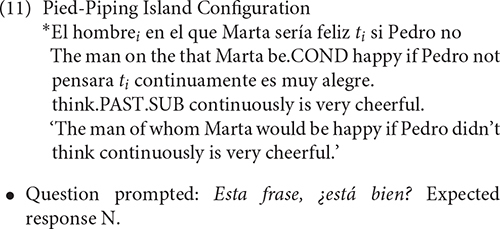

On the other hand, it is generally assumed that resumptive relative clauses do not engage movement and are interpreted through A-bar binding. For this reason, resumptive island configurations were coded as grammatical (12). In fact, the appearance of resumptive pronouns in island configurations is typically described as a last resort mechanism to rescue the derivation from the ungrammaticality.

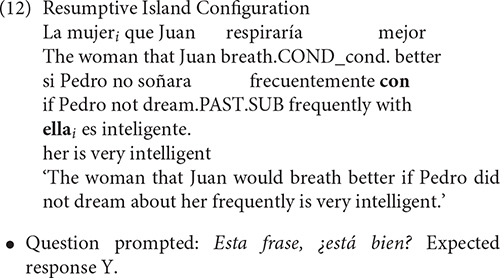

Finally, we also included in the experiment an island configuration with a relative clause formed through the Null-Prep strategy. This strategy was significantly produced by all L2 learners, and for this reason, we have decided to include it. This island configuration was *a priori* coded as ungrammatical, as a relative clause formed through Null-Prep.

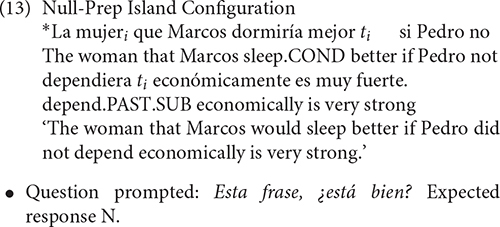

## Results Task 2: Self-Paced Gtj

**FIGURE 1:**
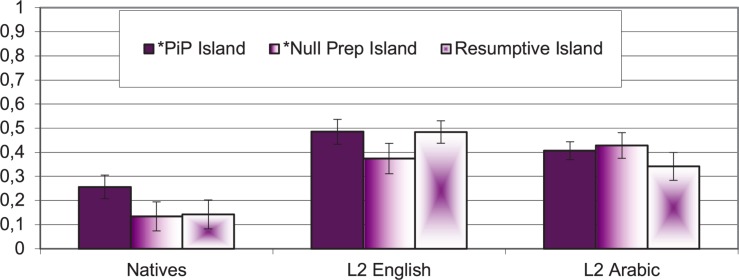
Acceptability results for island configurations in self-paced GJT (1 = grammatical, 0 = ungrammatical).

Accuracy was measured in average proportions, from 0 to 1 depending on the expected answer, where 1 indicated that the response given matched the codification made for that condition (correct response), and 0 indicated that the response given did not match the expected response (incorrect response). However, in order to understand the results independently from the aprioristic coding, accuracy was transformed into acceptability. This way, acceptability computes whether the participants judged the sentences as ok (“está bien”) or not ok (“no está bien”) regardless of the expected response. In these measurements, 0 means that the participant thought that the sentence was not ok, (not accepted) whereas 1 means that the sentence was ok (accepted). The average of these responses was calculated per structure and person. Figure 1 displays the acceptability averages per group and structure, with the Standard Error of the group.

The first interesting result is that native speakers overwhelmingly considered the sentences not ok, that is, ungrammatical. The L2 learners, on the other hand, do not seem to have robust intuitions regarding the acceptability of these sentences, accepting these sentences as adequate around 40–45% of the time. The proportions of acceptability responses were Arcsine transformed to account for their binomial distribution, and later submitted to a mixed-design repeated measures ANOVA with island structure (Pied-Piping, Null-Prep, Resumption) as a within-subjects factor, and group (native, L1 English, L1 Arabic) as a between-subjects factor. The Mauchly’s test indicated that the assumption of sphericity was not violated (ε = 0.991), and the within-subjects results revealed a mild main effect for structure [*F*(2, 120) = 3.706, *p* = 0.027, η*_*p*_*^2^ = 0.058], a main effect of group [*F*(2, 60) = 314.62, *p* < 0.001, η*_*p*_*^2^ = 0.328], but no significant interaction between structure and group (*p* > 0.05). The *post hoc* test for group indicated that the native speakers’ group was different from the two L2 learners’ groups (*p* < 0.001), and the two experimental groups did not differ between them (*p* = 1). We further explored the differences in structure and found that Pied-Piping was overall different from Null-Prep [*F*(1, 60) = 5.93, *p* = 0.018, η*_*p*_*^2^ = 0.090], and from the Resumptive condition [*F*(1, 60) = 4.61, *p* = 0.036, η*_*p*_*^2^ = 0.071]. However, when we carried out the within-subjects analysis independently for each group, the tests revealed that the main effect for structure only held in the native speaker group [*F*(2, 38) = 7.214, *p* = 0.002, η*_*p*_*^2^ = 0.275], but not in the learners’ groups (*p* > *0.1*). Likewise, only the native group distinguished between the Pied-Piping island condition and the Null-Prep island condition [*F*(1, 19) = 12.53, *p* = 0.002, η*_*p*_*^2^ = 0.397], and between the Pied-Piping island and the Resumptive island [*F*(1, 19) = 8.953, *p* = 0.007, η*_*p*_*^2^ = 0.320]; all the other contrasts were not significant (*p* < 0.01). To summarize so far, only the native speakers distinguished among the different types of islands, in favor of the gapped island, which was generally judged as more acceptable than the other two, against what has been reported in the theoretical literature.

## Discussion

In this study, we want to reflect on the (un)acceptability of island configurations in both L1 and L2, and its relation to the availability to *wh-*movement in these grammars. First, we will discuss the unexpected results from the native speakers and what these could mean for linguistic theory, and, in particular, for the theory of *wh-*movement, taking into account some psycholinguistic considerations. Later, we will discuss the data of the L2 learners and their implications for our views on the nature of interlanguage grammars.

The first main finding of this study is that native speakers, our control group, do not distinguish among island violations, and crucially, the resumptive pronoun does not improve the acceptance rates of these sentences. This is at odds with the traditional literature on island configurations and particularly with the assumed rescue effects of resumptive pronouns. Nevertheless, similar findings have been attested in McDaniel and Cowart (1999) with a relative acceptability judgment task for English relative clauses and islands; in Heestand et al. (2011) and Polinsky et al. (2013), studies devoted to the off-line and online comprehension of gapped and resumptive island constructions in native speakers of English, and in Alexopoulou and Keller (2007, 2013). In all of these experimental studies, it was found that when extracting from an island, the ungrammatical gapped condition was judged equal if not more acceptable than the supposedly “rescued” version with a resumptive pronoun. Our study corroborates these findings additionally for Spanish, as our native speakers found all extractions from island configurations unacceptable, both with a gap or a resumptive pronoun. Indeed, Spanish native speakers more often accepted the extraction with Pied-Piping from an island, which involves illicit *wh-*movement, than extractions from islands repaired with a resumptive pronoun. This is a novel result as, to our knowledge, Pied-Piping island configurations were not tested before, in English or in Spanish. It could be the case that the complexity of the extracted element (P+ *wh*-word) makes it more salient and/or more referential, and as such, it remains highly activated in memory (Just and Carpenter, 1992; Kluender, 1998; Hofmeister and Sag, 2010), making its integration in the discourse (d-linking) easier. These data would corroborate the main ideas of Hofmeister and Sag (2010) who propose that the unacceptability of island configurations goes beyond their syntactic nature, and is (also) motivated by the interaction of other cognitive constraints such as referentiality, saliency, d-linking, and/or the complexity of the filler phrase.

Granted, island sentences are difficult to judge, and require certain training and time, which the participants did not have. One of the reasons for choosing a timed GJT was to get the first, less conscious intuition about the structure. This would go with a generative view of language, which considers that real time construction of grammar sometimes loses grammar accuracy (Chomsky and Lasnik, 1993; Townsend and Bever, 2001), and against a view in which real-time processing can capture fine-grained distinctions (Phillips, 2003, 2006). In fact, this is not the only experiment which has failed to discover island sensitivity in processing experiments. For instance, Frazier and Clifton (1989) showed acceptance of gaps inside an island using speeded grammaticality tasks. Ferreira and Swets (2005) found dissociation between the production system and the comprehension system with respect to resumptive pronouns in island contexts. They found that native speakers of English judged these sentences as unacceptable in the grammaticality judgment task, but at the same time, they produced resumptives in islands in an oral experiment. Moreover, Ferreira and Swets (2005) further concluded that the “marginal” structure (resumptive island) takes more processing resources to produce, and participants found them harder to understand than a similar but grammatical construction. In their oral production experiment, the resumptive island construction was more often produced in the no time pressure condition than in the time-constrained condition, a result that the authors interpret as a sign of its costly nature, particularly with a RP. On the other hand, Chacón (2019) relates the appearance of RPs with long filler-gap dependencies that strain on working memory resources. That is, the RP appears as an anaphoric way to resolve the filler-gap dependency when the representation of the gap has failed. Similarly, Morgan and Wagers (2018) found that the production of RPs increases as the acceptability of a gap decreases. In any case, these proposals relate RPs with processing costs, implying that island configurations are not only a syntactic entity. This is also the position we take here. What seems to be clear from the experimental data gathered from GJTs is that RPs do not ameliorate island configurations; likewise, in this study, we failed to find an acceptability improvement of islands “repaired” by RPs, even in speakers who still produce RPs in relative clause formation, and whose native language (Arabic) accepts and requires RPs in these contexts. We interpret these results as a clear indication that islands, with gaps or with RPs, are not a purely syntactic phenomenon, and that using them as a means to determine the accessibility of L2 learners to UG is a moot point, as Belikova and White (2009) already concluded.

It must be acknowledged that the sentences included in the present experiment do not make complete sense, regardless of their grammatical status. In other words, these sentences are experimental in nature and are quite implausible, and we know that plausibility is a very relevant factor when interpreting sentences in real time (Traxler and Pickering, 1996; Pickering and Traxler, 1998; Pickering et al., 2000). Besides, there are several studies that have found that self-embedded sentences, such as the ones used in this experiment, are very hard to process due to memory capacity. This is the case because the reader needs to hold what has been read in memory for a long time, while also integrating new entities into the discourse (Lewis, 1996). Consequently, non-local dependencies are usually problematic not only for L2 learners (Dallas and Kaan, 2008) but also for monolingual native speakers (Gibson, 1998). The processing load of reading, memorizing and integrating meaning on-line makes comprehension and grammaticality judgments more difficult than in untimed tests. In the on-line GJT, there are factors such as word segmentation, memory or disruptions that play a significant role in quick decision making. The fact that paper and pencil experiments have found similar results indicates that all these factors are relevant and active when processing island constraints under no time pressure. Due to all this, we believe that island interpretation is a multifactorial matter, and that to isolate the most significant factors that contribute to their interpretability is very difficult, if not impossible. For instance, Kluender (1998) proposed that it is the interaction between verbal working memory and referential processing that explains the traditional dichotomy between strong and weak islands, and that, in the end, “wh-islands are essentially an interpretive problem” (Kluender, 1998:243).

These same considerations apply to the L2 learners’ processing, whose results are even less conclusive than those from the native speakers. Firstly, the production data indicates that for the most part, our L2 speakers form relative clauses through movement, particularly the English-speaking group. As for the Arabic group, 20.6% of their relative clauses are formed with a resumptive pronoun, and only three speakers constructed all relative clauses with the resumptive strategy, that is, without *wh-*movement, as hypothesized in H2. Assuming that Null-Prep relative clauses are also formed through movement, we can conclude that our L2 learners (except for those three Arabic speakers) know the rudiments of *wh-*movement in relative clauses, as hypothesized in H1. Still, they have very weak intuitions about the grammaticality of extractions from island configurations, and they tend to accept these (un)grammatical sentences between 40–50% of the time. Likewise, the L2 learners do not distinguish among the three types of extractions from islands, and similarly to the native speakers, do not have a preference for resumptive islands, that is, RPs do not improve their judgments about islands. One possible explanation for these results is to pose that native and L2 speakers alike tried to interpret resumptive islands through movement, as it would be the case with any other extraction. It is only after a processing failure that these sentences are interpreted through binding, and the RP is not able to repair the processing failure at this point. We favor an explanation — not incompatible with the previous one — which does not necessarily take these judgment data at face value. That is, it does not automatically condemn these resumptive structures and proposes that the speakers might not be judging the grammaticality of the sentence, but the plausibility, the naturalness, the depth of embedding, or simply that what we are measuring is the processability of this long sentence, and not its grammatical well-formedness.

Where do these data leave us in terms of the appropriateness of the methodology for our research purposes? How can we measure L2 knowledge of a phenomenon for which the native language does not provide a clear baseline? Crucially, our L2 learners, despite their weak intuitions, do not present the assumed contrast between gapped and resumptive islands, not even the learners whose native language presents resumptive pronouns in standard relative clauses (Arabic); but neither do native speakers. We suppose, then, that L2 learners are sensitive to the same type of processing and interpretative factors that native speakers are, even when their knowledge might still be in progress and present transfer effects, as found in Perpiñán (2015). This means that the L2 learners’ — and probably also the native speakers’ – processing might be somewhat dissociated from their grammatical knowledge, and even though the L2 grammatical representation might not be fully complete, the learners are able to grasp some of the interpretative and processing factors that condition the grammatical judgments on island configurations. In light of these results, this study contributes to the line of reasoning opened by Belikova and White (2009) and casts doubt on the suitability of assessing accessibility to UG by testing *wh-*islands, as it was typically done during the 90s. That is, if *wh-*islands are not a purely representational issue but an epiphenomenal one whose acceptability goes beyond grammatical well-formedness, for both native and non-native speakers alike, then GJTs on islands are not a reliable way to assess L2 grammatical knowledge. Still, they give us precious information on the way speakers interpret these sentences and whether L2 learners and native speakers resort to the same mechanisms while processing complex sentences.

## Data Availability Statement

The datasets generated for this study are available on request to the corresponding author.

## Ethics Statement

The studies involving human participants were reviewed and approved by the IRB, University of Illinois, Urbana-Champaign. Protocol Number: 08330. The patients/participants provided their written informed consent to participate in this study.

## Author Contributions

The author confirms being the sole contributor of this work and has approved it for publication.

## Conflict of Interest

The author declares that the research was conducted in the absence of any commercial or financial relationships that could be construed as a potential conflict of interest.

## Footnotes

1 Moroccan Arabic examples come or are adapted from Ennaji (1985).
